# Annual burden of disease in Nakaale, Karamoja: A descriptive, cross-sectional study

**DOI:** 10.1371/journal.pgph.0000222

**Published:** 2022-04-26

**Authors:** Leah J. Hopp, Ajwang Clementinah, Christopher J. Verdick, Agnes Napyo

**Affiliations:** 1 Orthodox Presbyterian Uganda Mission, Akisyon a Yesu Presbyterian Clinic, Nakaale, Nakapiripirit, Uganda; 2 Akisyon a Yesu Presbyterian Clinic, Nakaale, Nakapiripirit, Uganda; 3 Department of Public Health, Faculty of Health Sciences, Busitema University, Mbale, Uganda; University of California San Francisco, UNITED STATES

## Abstract

Even with global Sustainable Development Goals aimed at reducing poverty by reaching those furthest behind first and reducing inequalities within countries, regions like Karamoja continue to score low on many health indices. To understand the Karamoja context, we aimed to systematically describe the burden of disease in Nakaale, which is a hard-to-reach parish in Nakapiripirit District, through disaggregated data. This descriptive, cross-sectional study was conducted between January and December 2019. We relied on secondary data collected from all clients seen at Akisyon a Yesu Presbyterian Clinic’s outpatient department in Nakaale. Data was extracted from Uganda’s Health Management Information System records using Excel and exported to Stata for analysis. We labelled, categorised, and estimated frequencies and proportions for the variables. We estimated the means and medians for normally distributed or skewed variables respectively. A total of 14,685 observations, different diagnoses (n = 163) and residential locations or villages (n = 189) were extracted and included in the analysis. Nearly half (48.9%) of the clients were under the age of five years. Infectious diseases (42%), respiratory diseases (19%), and gastrointestinal and hepatic diseases (17%) were most commonly reported. While many clients (42%) reside in the sub-county where the health facility is located, a larger proportion (58%) came from further away, including 15% from outside of the local district. In conclusion, Akiyson a Yesu Presbyterian Clinic serves a very young population in a catchment area well beyond what is expected of a Health Centre II, in breadth of diagnoses, geographically, and in sheer numbers. Data gathered in this study will inform policy at the clinic, subcounty, and district levels enabling accurate health service delivery for the local context.

## Introduction

Even with global Sustainable Development Goals (SDGs) aimed to reduce poverty by reaching those furthest behind first and reducing inequalities within countries [1], hard-to-reach districts like Nakapiripirit [2], within low-income countries like Uganda [3], continue to score low on many health and sociodemographic indices [4]. In Sub-Saharan Africa, after the neo-natal period, nearly half of mortality in under-fives can be attributed to malaria, pneumonia, and diarrhoea [5], constituting an enormous burden of disease [6]. The high concentration of pneumonia and diarrhoea among poor and marginalised populations is a key marker of inequality both across and within countries [7]. Malaria is the most diagnosed illness in Uganda [8] followed by diarrhoeal diseases in under-fives in Uganda [9], with pneumonia being the fourth for under-fives in Uganda [10]. Karamoja is consistently among the highest transmission regions in Uganda for malaria [11].

The lack of quality data is problematic and often leads to treatment guidelines that are not sufficient for the local context [12]. Monitoring health inequalities is essential for improving health; yet, it requires various forms of disaggregated data [13]. A large data set was reviewed at a rural health facility where there is otherwise limited information; thus, this study serves as a baseline which will allow future local trends to be tracked. We aimed to describe the burden of disease in Nakaale, in order to inform the district in planning and accountability regarding provision of services that are a priority for the local population’s health needs. We also constructed an instrumental mapping of the geographical catchment area for our study setting within which our participants reside.

## Methods

### Study design

This descriptive cross-sectional design was conducted at Akisyon a Yesu Presbyterian Clinic (AYPC) in Nakaale, Nakapiripirit District, Uganda. The data was retrospectively collected from Health Management Information System (HMIS) Form 031, which is the standardised outpatient department (OPD) register used across all Ugandan public health facilities, including AYPC. We employed the use of a data extraction tool that was designed using Excel software [14] to collect data that was recorded from January 2019 to December 2019.

### Setting

AYPC is a private-not-for profit (PNFP) that is officially recognised by the government health care system as a Health Center (HC) II located in Nakaale parish, Loregae Sub-County, Nakapiripirit District, Karamoja sub-region, Uganda. Uganda’s health care system is decentralised and organised in hierarchies ranging from village health teams to HCII’s through HCIV’s up to referral hospitals [15]. In addition to government HCII provision of services [16], AYPC also offers dental care, basic obstetric services, laboratory, and pharmaceutical services. Each client pays a heavily subsidised standard user-fee during the registration process upon arrival which goes towards general operating costs of the health centre and covers all essential health services. The staffing level at AYPC exceeds what is expected of a HCII and includes the following: three clinicians (medical and clinical officers), two nurses, one enrolled midwife, three laboratory staff, one health educator, and 25 support staff (HMIS Form 107). This includes a community health team which conducts home-to-home health education sessions in the surrounding villages.

Northern Uganda, specifically the Karamoja sub-region, has uniquely endured decades of systemic marginalisation partially due to lengthy armed conflicts relating to cattle and its location in a semi-arid environment prone to climactic extremes. Infrastructure remains one of the top reasons why Karamoja is behind other regions. Improvements in the following areas are sorely needed: food security [4], agricultural knowledge in a post-nomadic setting, prevalence of those living in temporary dwellings [17], clean water accessibility and safe waste management [18], absenteeism of health workers [4], mean years of schooling [4], electricity [19], and accessible roads [20]. A combination of different factors relentlessly compromises the success of health in Karamoja. These include the break-down of the formal health-care system, the increased frequency of epidemics, the loss of adult family members to violent death, starvation, outward migration, the disruption of formal marriage structures, and the increasing problem of alcohol abuse [21].

### Participants and study size

In this study, we included all clients that were registered to receive health care services at AYPC’s OPD between January and December 2019. In this time period, a total of 15,818 observations were recorded in the HMIS Form 031 (OPD) register on variables including: unique OPD number, date, age, sex, residence, and diagnosis. However, 1133 observations had incomplete entries for some variables which were then excluded in the following order from this data set: six missing dates, five illegitimate OPD numbers, 176 missing ages, 35 missing sexes, 98 missing or unverifiable villages, and 813 missing or non-specific diagnoses. When analyzing missingness of the known characteristics of the 1133 excluded observations, it was assumed that unverifiable village names were rural since they could not be found on public maps and they were five kilometres or more from the clinic, since no local key informants knew their location. We therefore included 14,685 observations in our analysis. When analysing the different diagnoses, there are 19,480 illnesses total. The 866 observations of women attending antenatal care services at AYPC throughout 2019 were also not included in this data set because their information is recorded in a separate Antenatal Care register (HMIS Form 071) other than the OPD register.

### Measurement and variables

The age of clients was recorded in months for those under the age of one year and coded as zero years old, while those over the age of one year were recorded as completed number of years. The ages were further categorized into six groups in order to facilitate data analysis. We categorised age in years as <1, 1–4, 5–17, 18–35, 36–55 and ≥ 56 for comparability purposes. We categorised and labelled sex as ‘male’ and ‘female’. On-call hours are from 1701 hours until 0759 hours weekdays, including 24 hours on weekends and holidays. Consultations and referrals still occur on an emergency basis during on-call hours.

Each of the diagnoses were coded and categorised according to the International Classification of Diseases, Tenth Revision (ICD-10) [22] by clinicians. Diagnoses of malaria, brucellosis, and paratyphoid fevers, corresponding to ICD-10, were collectively categorised as being infectious diseases. The respiratory diseases category comprised of pneumonia, respiratory tract infections, and cough as the leading illnesses. Gastrointestinal diseases include amoebiasis, giardiasis, and gastroenteritis, among others. For the majority of cases seen, a laboratory investigation was conducted. Malaria is first investigated with a rapid test, then if that test is reactive, a blood smear is examined under the microscope to confirm malaria infection. Samples drawn to test for tuberculosis and measles are shipped to other laboratories for confirmation because AYPC’s laboratory does not conduct these tests. Some clients are diagnosed with multiple illnesses during the same visit to the health facility and are treated for these illnesses simultaneously.

Locations from which clients come to seek healthcare at AYPC were verified within parishes based on information from the Ugandan Electoral Commission [23], on the Land Conflict Mapping Tool website [24], and on the U.S. Geological Survey website [25]. There is no catchment area map for health service delivery available in Nakaale parish. This the first time in over ten years that a map of villages in Nakapiripirit District, and the surrounding area, has been generated in a systematic way with distances measured between the local villages and health centres. Residential locations that could not be externally verified were kept in the data set because of the paucity of data for this region. This study helped in the development of an accurate map [26]. Clients that seek healthcare at AYPC come from not only Nakaale parish, but also from other surrounding parishes, sub-counties, and even districts outside of Nakapiripirit. We categorised residences into “rural” and “urban” based on the definition from the National Population and Housing Census: “Urban centres include only the gazetted urban centres: cities, municipalities, town councils, and town boards” [27]. Some rural geographical areas like Nakaale village, in Nakapiripirit District, have not been documented in terms of map location, disease burden, and age distributions. With frequent political divisions and migration, this setting has changed extensively with the creation of additional parishes and villages. Information on these new geographical demarcations is not documented and there is no publicly available map to account for their existence. We relied on information collected at a PNFP HC.

If a client sought care at AYPC instead of their nearest designated health facility, then they were determined to have bypassed it to come to AYPC instead. There were no official referrals to AYPC. Some other clinics recommend their patients to follow-up at AYPC if they do not have the testing, medicines, or services prescribed at their facilities, yet they know that AYPC does. Based on a goal set for health centres in Uganda, residences were determined to be near to AYPC if they were geographically located within five kilometres [28].

### Data collection methods

A large data set was reviewed at a rural health facility where there is otherwise limited information; thus, this study serves as a baseline which will allow future local trends to be tracked. The entered data from HMIS Form 031 was routinely checked for completeness and accuracy. In this register, qualified clinicians recorded information on unique OPD number, date, age, sex, residence, diagnosis, medicines prescribed, screening for infectious diseases including tuberculosis, and laboratory investigations. Clinicians at AYPC have been trained on the importance of proper record-keeping and have been mentored by the in-charge, as well as their peers, on how best to validate information in the registers. AYPC collects client information using HMIS forms that have been approved by the Ugandan government in order to submit mandatory weekly and monthly reports. The records are also audited quarterly by the MOH for accuracy and completeness. Funding for AYPC depends on stringent documentation and internal quality control checks of all data.

Information was then collated using an Excel pre-designed, data-abstraction tool, and then formatted for data analysis in Stata, version 16.0 [29]. Normally distributed, continuous variables were summarised into means and standard deviations. Skewed variables were summarised into medians and interquartile ranges. Frequencies and percentages were calculated for categorical variables.

### Ethical approval and consent

Ethical approval to conduct this study was obtained from CURE–Children’s Hospital Uganda Research and Ethics Committee (reference no. CCHU-REC/05/021). Administrative clearance was given by the Nakapiripirit District Health Office and management of Akisyon a Yesu Presbyterian Clinic.

## Results

### Socio-demographics

A total of 15,818 observations were reviewed; however, 14,685 observations were included in this study analysis because they had the complete data on variables that were required for analysis. The median age of the clients was five years old (IQR 1, 25) (Table 1). More females (57%) than males sought care at AYPC. Nearly all of the clients (96.5%) were residing in a rural area and 59% had to travel a distance of five or more kilometres to access health care from AYPC. Three percent of overall clients are seen on an on-call basis, which are designated as emergencies. Though the majority of clients (67.1%) only presented with a single diagnosed illness, a third presented with either two or three illnesses at once. Most clients (66%) who sought care at AYPC during the year 2019 came in the months of July through December.

**Table 1:** Characteristics of clients and missingness at AYPC in Nakaale in 2019.

CHARACTERISTIC	Included n = 14,685	% Included	Incomplete n = 1,133	% Incomplete	Total n = 15,818	% Total
**Age (in years)**						
<1	3245	22.1	89	7.9	3334	21.1
1–4	3937	26.8	158	13.9	4095	25.9
5–17	2214	15.1	161	14.2	2375	15.0
18–35	3311	22.5	321	28.3	3632	23.0
36–55	1092	7.4	119	10.5	1211	7.7
≥ 56	886	6.0	109	9.6	995	6.3
Missing	0	0	176	15.5	176	1.1
**Sex**						
Female	8387	57.1	634	56.0	9021	57.0
Male	6298	42.9	441	38.9	6739	42.6
Missing	0	0	58	5.1	58	0.4
**Residence**						
Rural	14,173	96.5	989	90.5	15,198	96.1
Urban	512	3.5	41	3.6	553	3.5
Location unverified	0	0	36	3.2	36	0.2
No location provided	0	0	67	5.9	67	0.4
**Distance to AYPC**						
<5km	6040	41.1	463	40.9	6503	41.1
≥5km	8645	58.9	603	53.2	9248	58.5
Location unverified	0	0	36	3.2	36	0.2
No location provided	0	0	67	5.9	67	0.4
**Time of Reporting to HC**						
Working Hours	14,305	97.4	1072	94.6	15,377	97.2
On-Call Hours	380	2.6	61	5.4	441	2.8
**Number of Diagnoses**						
Single	9851	67.1	252	22.2	10,103	63.9
Double	4512	30.7	67	5.9	4579	28.9
Triple	322	2.2	3	0.3	325	2.1
Missing	0	0	811	71.6	811	5.1

After comparing excluded data separately, it was then added to the included data set to get a combined analysis [30] (Table 1). Almost all categories had a percentage change of less than one percent with the largest percentage change being less than four percent, confirming that by excluding the partial observations the results were not skewed. In the excluded data set, very similar results were found in the areas of female to male ratios seeking care at AYPC with 56% being female compared to 57.1% were female in the main study, 90.5% resided rurally in the excluded data set compared to 96.5% in the included data set, and clients coming from a distance of five kilometres or more from the clinic were 53.2% compared to 58.9%. Just as in the included data, a very small percentage of clients were seen during on-call hours. Even though most of the excluded data was due to missing diagnoses, those with known diagnoses maintained the trend of the majority presenting with a single diagnosis.

The only slight difference in data was found in the ages of clients where there was a larger percentage gap between the under-fives and those five years old and older with 21.8% of the observations in the excluded data being under-fives, 62.6% were five years old or older compared to 48.9% of the included data being under-fives and 51.1% being five years old or older. However, due to the relatively small number of excluded observations from the data set, when they were added into the overall total, there was no significant change in the results as 47% were under-fives compared to 48.9% in the included data alone.

### Diagnoses

Out of a total of 163 different diagnoses seen at AYPC in 2019, the top five most diagnosed diseases were: malaria (35%), pneumonia (11%), amoebiasis (6%), urinary tract infection (5%), and giardiasis (4%). The top five most common diagnosis categories seen at AYPC were: infectious diseases (42%), respiratory diseases (19%), gastrointestinal and hepatic diseases (17%), renal and urinary diseases (6%), and eye conditions (3%). Urinary tract infections made up 99% of the renal and urinary diseases category. Examples of infectious diseases include malaria, brucellosis, and paratyphoid fevers. Examples of respiratory diseases include pneumonia, respiratory tract infection, and cough. Examples of gastrointestinal diseases include amoebiasis, giardiasis, and gastroenteritis. A total of 60 percent of all cases seen at AYPC were diagnosed through laboratory investigations from blood samples, stool samples, urine samples, etc. comprising 33 out of a total of 163 diagnoses. Rapid tests used at AYPC are for brucellosis, diabetes, hepatitis B, HIV, hypoglycaemia, syphilis, and malaria. The other 26 diseases are confirmed with microscopy.

Diagnoses were categorised into 19 groups to supplement analysis as per ICD-10 (Table 2). Almost half (47.3%) of the diagnosis categories comprise less than one percent each of the total. Infectious diseases, mainly comprising of malaria, are by far the most commonly diagnosed illness at AYPC, followed by respiratory and gastrointestinal diseases. Because each person may have been diagnosed with more than one illness per visit, the total number of diagnoses is 19,840 instead of 14,685 for total number of observations.

**Table 2:** Diagnosis categories treated at AYPC in 2019.

Diagnosis Categories	n = 19,840	%
Emergencies and Trauma	335	1.7
Infectious Diseases	8381	42.2
Human Immunodeficiency Virus and Sexually Transmitted Infections	122	0.6
Cardiovascular Diseases	55	0.3
Respiratory Diseases	3706	18.7
Gastrointestinal and Hepatic Diseases	3358	16.9
Renal and Urinary Diseases	1093	5.5
Endocrine and Metabolic Disorders	85	0.4
Mental, Neurological, and Substance Use Disorders	42	0.2
Musculoskeletal Diseases	569	2.9
Blood Disorders	127	0.6
Oncology	1	0.01
Gynaecological Conditions	20	0.1
Obstetric Conditions	68	0.3
Nutrition	37	0.2
Eye Conditions	667	3.4
Ear, Nose, and Throat Conditions	349	1.8
Skin Diseases	505	2.6
Oral and Dental Conditions	320	1.6

**Fig 1:**
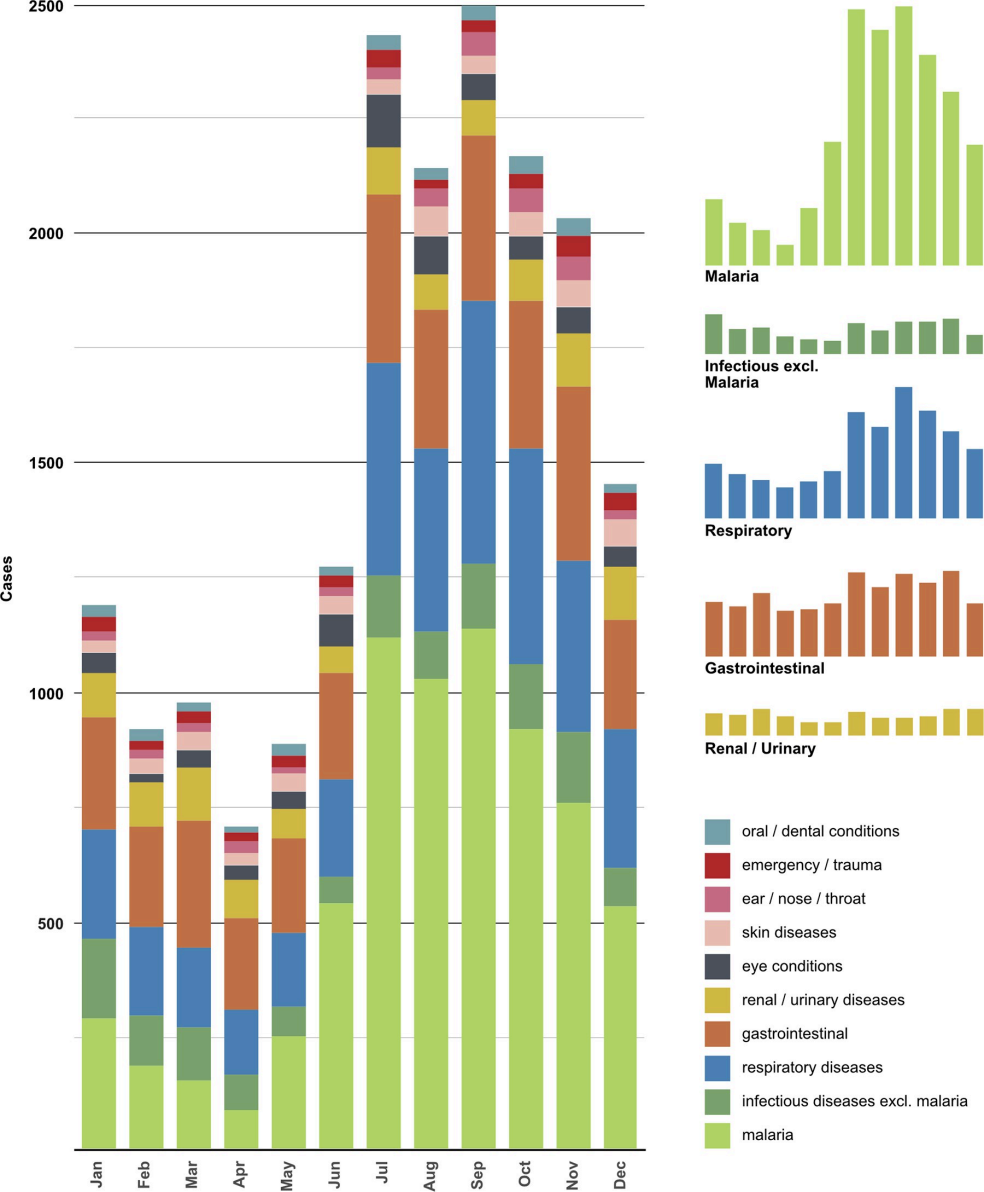
Diagnosis categories treated at AYPC in 2019. This graph was created using Affinity Designer Software [31]. Each bar in the graph indicates a different month of the year. Within each bar, the height of the sections indicates the number of cases for a particular diagnosis category. The trends can be followed throughout the year based on the different colours. The smaller bar graphs to the side pull out information for top diagnosis categories to highlight trends throughout the year.

The trend of cases from different diagnosis categories throughout the year can be tracked visually below (Fig 1). Though malaria is an infectious disease, because it represents the largest number of observations, it was included separately to clearly show differences in numbers throughout the year. Some categories from Table 2 were omitted from Fig 1 because they included too few cases to show visually in the bar graph, with 47% comprising less than one percent each of the total.

Nearly half (48.9%) of AYPC’s clients are under the age of five years old. Dividing the diagnosis categories into two groups delineated by the age of five years old shows that 58% of infectious diseases were diagnosed in the under-fives and 66% of respiratory diseases were diagnosed in the under-fives. For gastrointestinal diseases, only 46% of diagnoses were made in the under-fives. Looking at the data for patients seen by month and ages, for the first six months of 2019, under-fives are in the minority (30%). Infectious diseases, involving mostly malaria cases, were noticeably more represented during the months from July to November. Tabulating sex by diagnosis categories, one can see that females comprise more than half of patients seen in all categories except for endocrine and metabolic disorders.

### Mapping of residences

**Fig 2:**
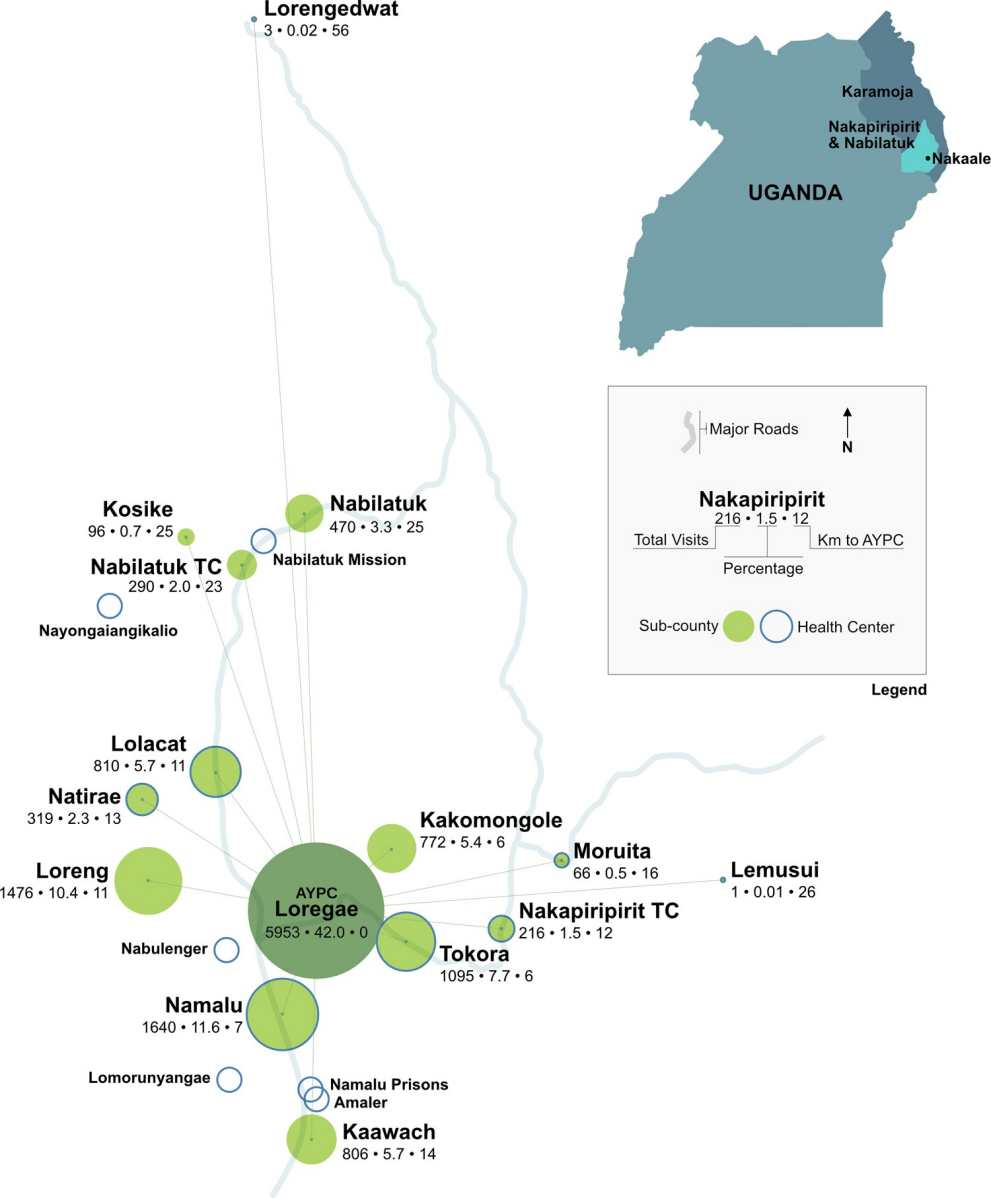
Total visits and kilometres from various sub-counties to AYPC in Nakaale in 2019. Created using Affinity Designer Software [31]. Sub-counties and health centres were plotted according to their visual location on a satellite base map from the U.S. Geological Survey using physical characteristics such as rivers, elevation, roads, and outlines of villages. Distances between each sub-county and AYPC, presented as the length of the lines, were measured relying on spatial distances from the U.S. Geological Survey [25]. The lines are arranged in a radial format with accurate angles representing where on the compass different locations lie in relation to AYPC. The number of clients seeking care at AYPC from sub-counties within Nakapiripirit and Nabilatuk Districts is shown by the size of the circle surrounding each sub-county name on the figure. The three numbers below the sub-counties refer to the total number of visits of clients from that location to AYPC, the percentage those visits comprise of the total observations for this paper, and the distance in kilometres that each sub-county is away from AYPC. Distances from AYPC are measured as the crow flies instead of the path someone might take along actual roads, which are shown on the infographic to spatially orient each sub-county. The thumbnail map of Uganda uses a base map from the Ugandan Electoral Commission’s website [25]. The GPS co-ordinates of AYPC are 1°51’25.8”N 34°36’55”E.

AYPC serves clients from 189 residential locations which are situated within 66 parishes, which are then grouped into 32 sub-counties and 10 districts. Each parish should have an HCII [32], yet AYPC serves clients residing in 10 districts; although, not the entirety of the population contained within those districts. Official sub-county boundaries, spatially represented on maps and listed within Ugandan districts, were referenced online [33]. The geographical relationship between AYPC and the neighbouring sub-counties is shown with distances represented by the length of the lines (Fig 2), including Nabilatuk District because up until the end of 2018, it was within the boundary of Nakapiripirit District. Many clients travel long distances to seek care at AYPC, including from 26km away within the same district, leaving aside those travelling from outside of the district. There are no other HC’s in the same sub-county as AYPC, yet there are ten in the same district. In this data set, 42 percent of clients from Loregae sub-county sought care at AYPC, leaving a sizeable portion (58%) travelling from other sub-counties which have their own health centres.

## Discussion

Nearly half (48.9%) of the individuals that seek healthcare at AYPC are under the age of five years old. We also find that there are more females than males. Most of the illnesses diagnosed at AYPC are infectious diseases, respiratory diseases, or gastrointestinal diseases.

People are more likely to bring their children under the age of five years old to seek care at the clinic instead of staying home with a sick child [34]. Those over the age of 56 years seek care the least, reflecting a lower investment in health for the elderly than children [34]. These numbers reflect the young population of Uganda with a high fertility rate and a low life-expectancy [35–37] The number of females seeking care at AYPC is higher for every age group, which coincides with demographics in other studies [38, 39], except for those under the age of one year where males made up 50.1% of the group, not just those in their reproductive years. Although women may be more likely to acknowledge sickness, inferior status in the family restricts their access to health care [40]. Analysing the number of females seen by other diagnosis categories shows that the trend holds for each category except for one: endocrine and metabolic disorders, and specifically lymphadenitis, but only marginally.

During a peak in the local rainy season, from June through October [41], infectious diseases were more routinely diagnosed; tellingly, the majority of this category consists of malaria cases [8]. One possible explanation of the trend towards more clients being seen during the second half of the year at AYPC could be because of seasonal variations in precipitation. One major influence on a higher number of clients being seen during the rainy season is that the number of cases of malaria increases dramatically during that time [42]. Under-fives were diagnosed more frequently during the rainy season of infectious diseases and respiratory illnesses, but older patients were more commonly diagnosed with gastrointestinal diseases during the same period, which was not expected [43].

In our study, we found there to be a high prevalence of infectious diseases, respiratory diseases, and gastrointestinal diseases. The findings of this study mirror trends found throughout Africa in the 21^st^ century showing that infectious diseases are responsible for large portions of mortality and morbidity patterns [44]. According to research done in northern Uganda, where conflict has also affected health care, major causes of mortality among children continue to be malaria, pneumonia, and diarrhoea which are managed by community health workers [45]. Another study in rural Uganda confirms that the burden of infectious diseases remains disproportionately high in low-income settings [46]. Respiratory diseases are still a leading cause of mortality in young children globally [47]. Acute respiratory infections still represent the most prevalent type of infectious diseases in many developing countries, including in Uganda, after malaria [48]. Worldwide, waterborne diseases are still a concern in resource poor countries [49]. In Uganda, amoebiasis and giardiasis are associated with poor and often marginalised communities and are often responsible for devastating illnesses especially in young children [9].

Poor nutritional status among children in Karamoja results from a combination of fever, malaria, and diarrhoea, etc. rather than a lack of food per se [50]. AYPC has intentionally not pursued incentivised treatment programmes nor project-based illnesses; instead, it focuses on the most common local illnesses. The laboratory at AYPC was the only one in the district to report stool microscopy. One of the reasons why there is such a breadth of diagnoses represented among this data set is because of having a medical officer on staff at that time. Because the cost of care at AYPC is very heavily subsidised, services are easily accessible to the general population, not just those who can afford private care. The most frequently diagnosed illnesses at AYPC are a good representation of the illnesses of the surrounding community [8]. The disease burden of many countries is shifting away from communicable, maternal, neonatal, and nutritional diseases (CMNN) to non-communicable diseases [51–53] This shift has not yet occurred at the rural village level in Nakaale as the most common diagnoses at AYPC are still in the CMNN category.

It is unusual for the majority of a HCII’s clients to reside over five kilometres from the facility; though, that is the case for more than half of AYPC’s clients. Most HCII’s are not expected to serve a population located beyond their parish [28]. Fig 2 shows how many other sub-counties are represented among client visits to AYPC, some of which are outside the local district. New sub-counties and parishes that have been created over the past ten years have added more infrastructure [4, 17–20] and accountability [4, 54] to the area, although AYPC remains the only HC in the sub-county. A possible reason for this is that AYPC is very consistent in maintaining their supply of medicines and laboratory supplies and thus are able to offer all their potential services, especially during the rainy season when some district roads become impassable. AYPC, as a private-non-for-profit health centre, is able to maintain sufficient levels of all supplies throughout the year due to solid financing and independent procurement avenues. Another factor drawing clients to AYPC from outside a five kilometre radius was the availability of a doctor on staff. It is not common practice for a higher level of health facility to refer patients to a lower level of care due to lack of resources or service and AYPC is only a HCII. There were no official referrals to AYPC; although, it is likely that some people may have self-referred, these were not two-way referrals [55]. Some other clinics recommend their patients to follow-up at AYPC if they do not offer the testing, medicines, or services necessary at their facilities, yet they know that AYPC does.

## Conclusion

Children under five years of age constitute nearly half (48.9%) among clients that seek health care services at AYPC. Clients that seek health care at AYPC reside in various locations that are beyond the predetermined AYPC catchment area. The most common diagnoses made among the clients attending AYPC in Nakapiripirit District include malaria, pneumonia and amoebiasis. The largest local disease burden occurs between the months of July and November. We therefore recommend that while the District Health Office and AYPC plan for health services, more emphasis needs to be put on those affecting children under five and also increasing health supplies for AYPC during the months of July through November.

### Recommendations for further study

A qualitative study with interviews of key informants would allow for further explanation of why clients sought care at AYPC instead of from their nearest health centre. Another idea for a focus group could expound upon the health seeking habits of the local population. Studying this data set analytically will yield more information to add to the local body of knowledge which informs health programming decisions. Further verification of the GPS locations of villages in Nakapiripirit District would aid all the local health centres in understanding this setting better. Another valuable avenue of inquiry would be regarding common factors contributing to hygiene and sanitation illnesses. We recommend further studies in this setting to attempt to explain why more females and so many children seek healthcare. Gathering comparative data from other HC’s in the district would give valuable information and lend further insight into the local burden of disease.

### Strengths and limitations

To the best of our knowledge, this paper is the first to profile this context in terms of sociodemographic profiles, the burden of disease, and geographical distribution. The data represented in this paper are actual numbers, not projections nor estimates nor summaries of trends applied from other regions. This paper will act as a point of reference for subsequent research that will be conducted in this setting. Some of the observations (8%) were incomplete as a result of documentation errors in the HMIS register, which were subsequently excluded and thus some unique characteristics of the study area could not be fully captured. The trends and conclusions in this paper were not affected by the missing information excluded. Variables on health care seeking behaviour were not included in this study because the data is not routinely collected in the OPD register. This study did not differentiate between new patients and those returning to the health centre for review which could give a more accurate picture of how often people become sick.

## Supporting information

S1 Data Data set analysed for this study. (XLSX)

S2 Data Full data collected from AYPC in 2019. (XLSX)
